# Recent advances in understanding skeletal muscle ageing: Functional, morphological and omics perspectives

**DOI:** 10.1113/EP093857

**Published:** 2026-05-10

**Authors:** Oscar Horwath, William Apró

**Affiliations:** ^1^ Department of Women's and Children's Health Karolinska Institutet Stockholm Sweden; ^2^ Department of Physiology, Nutrition and Biomechanics The Swedish School of Sport and Health Sciences Stockholm Sweden

**Keywords:** DNA methylation, muscle atrophy, myosin heavy chains, plasticity, sarcopenia

## 1 INTRODUCTION

Connections link a sequence of three related research papers. The central article which links the other two papers has been published in Experimental Physiology. In a Connections article, an author (or authors) of the central article outlines its principal novel findings, tracing how they were influenced by the first article and how the central article has contributed to the developments made in the third article. The author(s) may also speculate on the direction of future research in the field. Connections articles aim to set the research in a wide context.

Over the course of the lifespan, skeletal muscle undergoes marked changes in mass, function and form. In humans, it grows steadily from birth into early adulthood, remains rather stable through midlife, and then, typically around the fifth decade, begins a noticeable decline. In severe cases, this loss can progress into sarcopenia, a condition linked to frailty, falls, nursing home admissions and reduced quality of life. Fortunately, this trajectory is not fixed. Interventions such as resistance exercise training can slow and even reverse muscle loss. While older adults may show a somewhat reduced response compared to younger individuals, they can still achieve meaningful gains in strength and mass well into later life.

For many years, researchers have studied skeletal muscle from older adults to understand how ageing affects the tissue at the cellular and molecular level. However, isolating the effect of ageing itself is challenging and it is difficult to separate this from lifestyle factors common in older adults, such as inactivity, adiposity and medication use. These factors are closely linked to an ‘aged’ lifestyle but do not reflect the intrinsic effects of chronological age, making it difficult to disentangle true age‐related changes. This is particularly true for a highly plastic tissue such as skeletal muscle, which is known to be highly influenced by, for example, inactivity. Despite this complexity, recent studies have advanced our understanding of aged muscle. The three publications outlined in this Connections article provide valuable insights into how muscle size, shape and function are altered with age and how aged muscle responds to increased mechanical load. Together, they cover a wide spectrum of insights, ranging from observations at the cellular level in humans, moving to an intervention in mice that combines deep molecular and computational approaches.

The first study, from Dr Sundberg's team, provides a comprehensive synthesis of how ageing affects the contractile properties of human muscle fibres, presenting data accumulated over the past 25 years from skinned fibre preparations (Grosicki et al., 2022). In this technique, the muscle cell membrane is removed mechanically or chemically, enabling precise control of the intracellular environment, including calcium concentrations, while assessing contractile function. Importantly, the analysis distinguishes between the two principal fibre types in human skeletal muscle: type I (slow‐oxidative) and type II (fast‐glycolytic), which differ markedly in metabolism and contractile behaviour. While the relative abundance of these fibre types can shift with age, analysing them separately is key for understanding fibre‐specific adaptations. Strikingly, the findings reveal that ageing does not affect all fibres equally. Type I fibres appear largely preserved, showing minimal decline in contractile function or size. In contrast, type II fibres exhibit substantial reductions in size, which emerge as the primary determinant of their reduced force production. This indicates that the intrinsic contractility of these fibres is maintained, and that the observed functional decline is driven predominantly by atrophy (Grosicki et al., 2022). Together, these results provide evidence for selective fibre atrophy, where ageing disproportionately impacts type II fibres.

An important unresolved question is the extent to which the observed fibre‐specific alterations reflect intrinsic ageing, rather than differences in lifestyle factors. The cohorts included in previous work span a broad spectrum, from sedentary individuals to highly trained older athletes, introducing variability in physical activity, body composition, diet and medication use, factors that are not inherent to the ageing process itself and therefore may confound interpretation. To address this, our research group used a cross‐sectional design comparing two groups of healthy men separated by five decades in age. Importantly, the groups were closely matched for key lifestyle and metabolic characteristics, such as body mass index, exercising habits, medical use and metabolic responses to feeding, providing a robust model for isolating the effects of chronological ageing on skeletal muscle (Horwath et al., 2025).

Muscle cross‐sections prepared from the vastus lateralis muscle revealed a consistent pattern. Type I fibres were largely preserved, whereas type II fibres exhibited pronounced deficits, including reduced fibre size, altered morphology and a lower abundance of satellite cells and capillaries. In addition, there was an increased global presence of denervation markers and fibre type grouping, indicating ongoing cycles of denervation and reinnervation (Horwath et al., 2025). Together, these findings demonstrate that selective vulnerability of fast‐twitch fibres is an intrinsic feature of human muscle ageing, rather than simply a consequence of lifestyle. At the same time, they raise an important mechanistic question: why are type II fibres particularly susceptible to age‐related decline? Addressing this will be critical for understanding, and ultimately mitigating, sarcopenia. We therefore strongly encourage the field to move in the direction of greater cellular granularity trying to resolve these mechanisms.

Another feature of ageing muscle is a reduced capacity to adapt to anabolic stimuli. While resistance exercise reliably induces hypertrophy in young, this response is blunted with age. This suggests that the intrinsic deficits observed at the fibre level may translate into an impaired ability of aged muscle to remodel in response to increased load. To investigate the mechanisms underlying this reduced adaptive capacity, Koopmans and colleagues used a rodent model of mechanical overload (Koopmans et al., 2026). The authors then applied an array of analytical approaches, combining global transcriptomics with a modified doxycycline‐inducible model called HSA‐GFP, which allows characterisation of transcriptional and epigenetic profiles specifically in resident myonuclei. At baseline, aged muscle displayed elevated expression of genes linked to ribosome biogenesis and mechanistic target of rapamycin complex 1 (mTORC1) signalling, a paradoxical finding given the central role of these pathways in promoting protein synthesis. This may reflect dysregulation of mTORC1 and impaired proteastasis, an emerging hallmark of ageing skeletal muscle.

**Connected Articles**

- Grosicki, G. J., Zepeda, C. S., & Sundberg, C. W. (2022). Single muscle fibre contractile function with ageing. *The Journal of Physiology* 600(23), 5005–5026.
- Horwath, O., Moberg, M., Edman, S., Philp, A., & Apró, W. (2025). Ageing leads to selective type II myofibre deterioration and denervation independent of reinnervative capacity in human skeletal muscle. *Experimental Physiology* 110(2), 277–292.
- Koopmans, P. J., Jones, R. G., 3rd, Cabrera, A. R., Morena, F., Greene, N. P., McCarthy, J. J., Ismaeel, A., Wen, Y., & Murach, K. A. (2026). The age‐dependent resident myonuclear multi‐omic response to an acute skeletal muscle hypertrophic stimulus in mice. *Advanced Science*, 13(25), e21633.

In response to mechanical overload, although many aspects of the transcriptional response were preserved, aged muscle exhibited a diminished induction of pro‐hypertrophic regulators, including *Mybph* and *Myc*. These differences were accompanied by alterations in myonuclear DNA methylation of the same genes, providing strong support to epigenetic regulation as a key mechanism underlying reduced adaptive capacity (Koopmans et al., 2026). In addition, transcripts associated with the neuromuscular junction, such as *Ncam1* and *Runx1*, showed a more robust induction in adult compared with aged mice. This suggests that aged muscle may have a reduced capacity to remodel the neuromuscular junction in response to increased loading, with potential implications for establishing efficient nerve–muscle communication required for strength gains. One possible explanation is that this attenuated response reflects a greater burden of denervation in aged muscle. Alternatively, it may arise from shifts in the contribution of non‐myofibre cell populations. Indeed, using cell deconvoluted data, the authors suggest a reduction in glial cells, which are known to play a key role in maintaining integrity of the neuromuscular junction under conditions of altered neural input. These findings emphasize that the molecular response to loading in aged muscle is not only altered at the level of myofibres but also shaped by changes in the cellular composition. This underscores the need to move beyond the traditional ‘myofibre‐centric view’ and consider the potential contributions of other cell types to blunted muscle adaptation in ageing.

In summary, these studies highlight the intrinsic vulnerability of human type II fibres with ageing and identify mechanisms underlying the reduced adaptability to mechanical loading in ageing mice. Future research should assess the translatability of these findings to humans, particularly the loss of epigenetic regulation and its conservation across species. Additionally, further fibre type‐specific investigations are warranted. Together, such efforts will advance our understanding of the fundamental mechanisms driving age‐related decline and inform the optimisation of strategies aimed at preventing sarcopenia.

## AUTHOR CONTRIBUTIONS

Both authors have read and approved the final version of this manuscript and agree to be accountable for all aspects of the work in ensuring that questions related to the accuracy or integrity of any part of the work are appropriately investigated and resolved. All persons designated as authors qualify for authorship, and all those who qualify for authorship are listed.

## CONFLICT OF INTEREST

None declared.

## FUNDING INFORMATION

None.
